# Combining Ability and Heterotic Patterns of Tropical Early-Maturing Maize Inbred Lines under Individual and Combined Heat and Drought Environments

**DOI:** 10.3390/plants11101365

**Published:** 2022-05-20

**Authors:** Alimatu Sadia Osuman, Baffour Badu-Apraku, Beatrice Elohor Ifie, Charles Nelimor, Pangirayi Tongoona, Ebenezer Obeng-Bio, Benjamin Karikari, Eric Yirenkyi Danquah

**Affiliations:** 1 West Africa Center for Crop Improvement (WACCI), University of Ghana, PMB 30 Legon, Accra, Ghana; asosuman@wacci.ug.edu.gh (A.S.O.); bifie@wacci.ug.edu.gh (B.E.I.); ptongoona@wacci.ug.edu.gh (P.T.); edanquah@wacci.ug.edu.gh (E.Y.D.); 2 International Institute of Tropical Agriculture (IITA), PMB 5320, Ibadan 200001, Nigeria; 3 Crops Research Institute (CRI), P.O. Box 3785, Kumasi, Ghana; 4 Savanna Agricultural Research Institute (SARI), P.O. Box TL 52, Tamale, Ghana; cnelimor@yahoo.com; 5 Cocoa Research Institute of Ghana, (CRIG), P.O. Box 8, New Tafo-Akim, Ghana; obengbio2000@gmail.com; 6 Department of Crop Science, Faculty of Agriculture, Food and Consumer Sciences, University for Development Studies, P.O. Box TL 1882, Tamale, Ghana; benkarikari1@gmail.com

**Keywords:** combining ability, DArTseq, heat stress, combined heat and drought, *Zea mays*

## Abstract

Information on combining ability and heterotic patterns of multiple stress-tolerant inbred lines are fundamental prerequisites for devising appropriate breeding strategies for the development of climate-resilient maize hybrids. In the present study, we evaluated 150 single cross hybrids derived from the North Carolina Design II (NCD II) along with six commercial checks under terminal drought stress (TDS), heat stress (HS), and combined drought and heat stress (CHDS)conditions. The objectives of the study were to: (i) determine the combining ability of the inbred lines and identify the best testers across the stresses; (ii) classify the inbred lines into heterotic groups (HGs) based on the general combining ability of multiple traits (HGCAMT) and sequencing-based diversity array technology (DArTseq) and (iii) assess the performance and stability of the lines in hybrid combinations. The inbred lines showed significantly (*p* < 0.01 and *p* < 0.05) positive and negative general combining ability (GCA) and specific combining ability (SCA) effects for grain yield (GY) and most other measured traits. The inbred line TZEI 135 displayed relatively larger positive GCA effects for GY when mated either as male or female and was identified as the best tester. TZEI 135 × TZEI 182 was identified as the best single-cross tester across environments. Results of the assessment of the relative importance of GCA and SCA effects revealed the predominance of additive gene action over the non-additive. Six HGs of inbreds were identified using the HGCAMT and three, based on the DArTseq marker genetic distance method, were the most efficient. The best hybrids in this study significantly out-yielded the best checks by 21, 46, and 70% under CHDS, HS, and TDS, respectively. These hybrids should be extensively tested in on-farm trials for possible commercialization in sub-Saharan Africa.

## 1. Introduction

Maize (*Zea mays* L.) is an important cereal crop cultivated globally for multiple purposes, such as staple food, livestock feed, edible oil, biofuel, and raw material for the synthesis of various industrial products [1]. In Sub-Saharan Africa (SSA), maize serves the dietary needs of nearly 50% of the population [2]. Even in the fringes of the Sudan Savanna of West Africa where millet and sorghum are predominantly cultivated, maize has made significant inroads as a major source of food and livelihood for the majority of the farming households. Despite the crucial role that maize plays in SSA, its production and average grain yield (GY) per hectare are low because of recurring climatic stresses during the cropping season. Drought and high temperature are among the principal stresses that impede maize growth and cause huge GY losses. About 40% of the annual maize yield loss in SSA has been attributed to drought stress (DS) [3]. NeSmith et al. [4] reported that only 10% of the potential yield of maize can be realized when DS occurs during the reproductive stage of maize growth. Heat stress (HS) at this same stage of maize development can denature and render the pollen infertile, leading to poor seed set and ultimately poor yield [5]. The effects of DS and HS on maize growth and productivity are compounded by the rapidly changing climate that promises to aggravate the frequency and severity of both stresses. In the face of climate change, DS and HS are increasingly likely to occur simultaneously during the growing season and the consequences are expected to be profound on maize cultivation in SSA [6]. The combined effect of HS and DS (CHDS) on the GY of maize has been reported to be higher than the effects of each stress alone but lower than their sum [6,7,8]. Global crop yield losses caused by CHDS alone translated to approximately US 200 billion annually between 1980 and 2012 [9]. The growing losses represent a major impediment to the food and economic security of millions of smallholder farmers who depend on maize for their livelihood. Thus, it is critical to develop and screen maize genotypes for tolerance to DS and/or HS at the reproductive stage. To achieve this, managed DS experiments are designed to expose breeding materials to moisture deficit at flowering and early grain filling stages with a resultant yield loss of 40–90% [2]. The optimal day temperatures for maize range between 25–33 °C, while night temperatures range between 17–23 °C [2]. Therefore, planting time for screening for HS tolerance should be planned in such a way that the anthesis to early grain-filling stages coincides with the maximum and minimum temperature rises >35 °C and 23 °C, respectively for at least two weeks [10].

Unlike the drought tolerance maize breeding which has achieved remarkable success, explicit breeding for HS tolerance began only recently in SSA [1,2]. Indeed, given the expected co-occurrence of DS and elevated temperatures on farmers’ fields, it is important that breeding lines are selected for tolerances to both stresses [6]. Moreover, it has become increasingly clear that the demand for maize varieties/hybrids that are tolerant to the combined effect of both DS and HS will increase dramatically especially in the Sahel of West and Central Africa (WCA) as global climate change increases [6].

A thorough understanding of the general combining ability (GCA) and specific combining ability (SCA) of a breeding population is critical for the adoption of the most appropriate breeding strategy to improve the traits of interest [11]. While GCA is associated with the additive gene action or breeding value, SCA is indicative of the non-additive gene action modulated by dominance and epistatic gene effects including aberrations due to genotype-environment interactions. When additive gene action predominates, the implication is that genetic gains from selection could be maximized through population improvement methods such as the S_1_, full-sib, and half-sib family selection methods while a preponderance of the non-additive gene action indicates that outstanding varietal hybrids could be identified by testing the inbred lines in various hybrid combinations [12]. An ultimate goal of a plant breeder is to develop hybrids with high heterosis over their parents for a target trait. Brieger [13] showed that in maize, a higher degree of heterosis can be obtained when the parents from which the hybrids are derived belong to opposing heterotic groups. A heterotic group (HG) refers to a collection of germplasm that when crossed with germplasm from another group, gives rise to desirable heterosis (on average) compared to the crossing of pairs of the same group [14]. Akinwale [15] defined a heterotic pattern as a specific pair of two HGs that show high heterosis and/or superior hybrid performance in their cross. Thus, before starting a hybrid maize breeding program, it is critical to classify available genetic materials into distinct heterotic groups/patterns. Moreover, to get the most out of heterosis for enhanced genetic gain in hybrid maize breeding programs, the availability of efficient testers for effective discrimination and heterotic grouping of inbred lines is a crucial requirement. According to Badu-Apraku et al. [2], a good tester should be able to discriminate between lines within its HG and those from opposing groups. Additionally, a good tester should be able to represent the inbreds of its group and should be effective in assigning other inbreds into distinct HGs based on Line × Tester analyses.

The North Carolina Design II is one of the prominent mating designs in plant breeding for classifying inbred lines into appropriate HGs and identifying desirable testers for use in hybridization [15]. Conventionally, the methods used in assigning breeding lines into distinct HGs are based on either SCA or a combination of SCA and GCA effects of a single trait, i.e., GY. However, genetic progress based on the direct selection for GY alone is usually very slow due to its complex nature and low heritability, especially under abiotic stress conditions [15]. To address this shortcoming, Badu-Apraku et al. [16] proposed a method that integrates GCA effects of multiple traits-heterotic groups based on the GCA of Multiple Traits (HGCAMT). Furthermore, recent advances in DNA-based molecular marker technology including genotyping-by-sequencing derived single nucleotide polymorphism (SNP) and sequencing-based diversity array technology (DArTseq) markers offer a somewhat cheaper, flexible, faster, and smarter alternative for identifying HGs using genetic distance (GD) [15]. Akinwale [15] opined that HGCAMT and molecular marker-based approaches of assigning maize inbreds into HGs give different results and that the efficiency of these methods differ depending on the mode of gene action prevalent for the trait(s) under study. Badu-Apraku et al. [16] compared the efficiencies of the two methods for defining HGs among early-maturing yellow maize inbreds and observed the superiority of the HGCAMT method over the SNP-based GD methods. In another study, Badu-Apraku et al. [17] attempted to classify 14 early maturing yellow-endosperm QPM inbred lines into HGs using HGCAMT and SNP-based GD methods and reported that the former was the most efficient.

Breeders at the International Institute of Tropical Agriculture (IITA) in collaboration with the National Agricultural Research Institutes (NARS) of WCA have, over the years, developed several DS-tolerant inbred lines. Efforts at determining the mode of gene action controlling the inheritance of GY and other traits in these inbred lines have yielded conflicting reports. For example, previous studies [18,19] have demonstrated the importance of both additive and non-additive gene actions but with a greater preponderance of the former over the latter in the inheritance of GY under DS. Badu-Apraku et al. [20] examined the combining ability of 30 extra-early maturing maize inbred lines under DS and found that the general combining ability (GCA) effects accounted for 70% of the total genetic variation, which further confirmed the importance of additive gene action in the inheritance of GY under DS. In contrast, the predominance of non-additive genetic effects has been reported in other studies [21,22]. Considering these conflicting reports, additional studies involving inbred lines derived from diverse genetic backgrounds could contribute to the understanding of the mode of gene action controlling GY under DS. Because of the emerging threats of extreme temperatures to maize cultivation in SSA, several early maturing inbred lines with tolerance to CHDS have been developed at IITA. Compared to DS, there have been few genetic studies focusing on the combining ability of maize under HS and CHDS. Recently, Nasser et al. [23] studied the GCA effects of 20 early maturing inbred lines from the IITA-Maize Improvement Program and SCA of the derived hybrids under CHDS and stress-free environments. It was noted that the GCA and SCA effects for GY and other measured traits were significant, but GCA effects were greater than the SCA effects under both CHDS and stress-free environments. However, the combining ability and HGs of a large number of the IITA’s newly developed DS and CHDS–tolerant inbred lines have not been determined to enable their efficient use to maximize heterosis breeding of hybrids in the tropics. In the present study, we inter-crossed a set of 30 early maturing white and yellow maize endosperm inbred lines derived from diverse germplasm sources to generate 150 single cross hybrids using the North Carolina Design II mating scheme. Our objectives were to:

- Assess the importance of general combing ability (GCA) and specific combining ability (SCA) for grain yield and other key agronomic traits in a set of newly developed inbred lines under independent and combined drought and heat stress conditions;
- Identify inbred and hybrid testers across test environments;
- Assign the inbred lines into heterotic groups based on the GCA of multiple traits (HGCAMT) method and DArTseq markers, and assessed the performance and stability of the hybrids under the test environments.

## 2. Materials and Methods

### 2.1. Genetic Materials and Crossing Scheme

Thirty DS- and CHDS-tolerant early maturing inbred lines were selected for this study based on preliminary evaluation (data not presented) under DS and CHDS. These included 15 of each white and yellow endosperm maize inbred lines derived from diverse sources. First, some of the inbred lines were extracted from broad-based populations derived from exotic and local germplasm with broad adaptation to dry and hot climates identified based on many years of extensive testing in the savannas of WCA. The second set of inbred lines was developed through crosses involving a broad-based drought-tolerant population and elite *Striga* resistant inbred lines as well as hybridization between a drought-susceptible population and drought-tolerant inbred lines. In addition, some of the lines were derived from two IITA bi-parental populations. Detailed information on the methodology and strategies adopted for the development of the inbred lines as well as the genetic backgrounds of the broad-based source populations, *Striga* resistant, drought-tolerant, and susceptible inbred lines, and the IITA-biparental populations have been extensively described in a previous study [24]. The 30 inbred lines selected for this study were grouped into six sets of five lines based on endosperm color and the strategy adopted for developing the lines. The inbred lines were intercrossed using the North Carolina II (NCD II) mating design such that each set of crosses involved inbred lines with different genetic backgrounds to maximize heterosis. Each line was used as a male in one set and as a female parent in another set. A total of 150 F_1_ progenies (single cross hybrids) were generated and six commercial hybrids with good levels of tolerance to DS and CHDS conditions were included as checks for field evaluations. The pedigree information of the parental lines, their reactions to the stresses as well as the sets into which they were placed, and a description of the checks have been presented in Supplementary Table S1.

### 2.2. Field Evaluation and Agronomic Management

The 150 NCD II derived hybrids along with six commercial checks were evaluated at the Manga Station of the Savannah Agricultural Research Institute, Ghana (11°00.977 N, 000°15.912 W 252 m altitude) under terminal drought stress (TDS), HS, and CHDS conditions during the growing and dry seasons of 2018 and 2019, respectively. The hybrids were also evaluated under CHDS at the IITA experimental site at Kadawa, Nigeria (11°39′ N, 8°27′ E, 500 m altitude) between February and June in 2018 and 2019. A 12 × 13 alpha lattice design with 2 replications was used in all the experiments. At all locations, three seeds were planted in single-row plots, each 3 m long. Inter and intra row spacings were 0.75 m and 0.4 m, respectively. Two seedlings were maintained per hill to achieve a density of 66,667 plants/ha. At Manga, experiments under TDS were planted in the middle of September each year to induce natural DS at the reproductive stages in November. Trials under HS and CHDS were planted on opposite blocks separated by 10 m to prevent spillover of irrigation water. The trials were planted on the same day in the second week of February to ensure that the flowering and grain filling stages coincided with the peak temperature in April (day/night 39.4/18.2 °C in 2018, and 40.1/26.1 °C in 2019) (Supplementary Table S2). Similarly, the CHDS experiments at Kadawa were planted in mid-February in 2018 and 2019. For each year, day and night temperatures were lowest during planting in February and highest in April during the reproductive stages (Supplementary Table S2). For each experiment, basal fertilizer was applied at the rate of 60 kg each of N, P_2_O_5,_ and K_2_O ha^−1^ (15:15:15) at planting. Three weeks later, 60 kg ha^−1^ of urea was applied as a top dressing. The HS and CHDS blocks were supplied with irrigation water using a drip irrigation system at Manga and a furrow irrigation system at Kadawa. At both locations, irrigation was suspended on the CHDS block at 34 days after planting to induce DS at elevated temperatures prior to and during flowering and grain filling periods. Irrigation was resumed 10 days after completion of grain filling but was conducted if and when the plant showed signs of severe leaf rolling and wilting during the early mornings. The HS block received irrigation water from planting to physiological maturity. At both Manga and Kadawa, the flowering and grain filling periods generally occurred in April when day and night temperatures were relatively high in both years (Supplementary Figure S1a,b). Pre- and post-emergence weeds were controlled using atrazine and Gramoxone, supplemented with hoe weeding.

### 2.3. Traits Measured

Days to anthesis (AD) and silking (SD) were recorded for each plot when 50% of the plants had their pollen shed and silks extruded, respectively. The anthesis-silking interval was computed by subtracting AD from SD. Plant and ear heights (PLHT and EARHT) were recorded on representative plants of each plot at physiological maturity. PLHT was measured from the base of the plant to the tip of the tassel while EARHT was measured from the base of the plant to the node bearing the uppermost ear. Plants with tassel blasting (TB) and leaf firing (LF) symptoms were counted for the HS and CHDS plots. Ear rot (EAROT) was counted on each harvested plot and converted to percentages. Plant aspect (PLASP) was rated based on the assessment of the general architecture of plants in a plot as they appeared by sight using a scale of 1–9 where 1 = excellent overall-physical-appearance, 2 = very good overall-physical-appearance, 3 = good overall-physical-appearance, 4 = relatively good overall-physical-appearance, 5 = acceptable physical appearance, 6 = relatively poor overall-physical-appearance, 7 = poor overall-physical-appearance, 8 = very poor overall-physical-appearance, and 9 = extremely poor overall-physical-appearance [2]. Husk cover (HC) was visually rated using a scale of 1–9 where 1 means a tightly arranged husk extending to the ear tip and 9 represents a loose and exposed ear tip. The stay-green characteristic also termed as the leaf death characteristic (LDC) was recorded on a scale of 1–9 where 1 = 10% dead-leaf-area, 2 = 20% dead-leaf-area, 3 = 30% dead-leaf-area, 4 = 40% dead-leaf-area, 5 = 50% dead-leaf-area, 6 = 60% dead-leaf-area, 7 = 70% dead-leaf-area, 8 = 80% dead-leaf-area, and 9 = 100% dead-leaf-area. Ear aspect (EASP) was visually assessed for texture, size of the ears, extent of grain-filling and uniformity of the ear size using a scale of 1 to 9, where 1 = outstanding ears with large cobs, disease and insect free, fully filled grains and uniform ears, 2 = excellent disease and insect free ears with fully filled grains, one or two irregularities in cob size, 3 = good disease and insect free ears, fully filled grains with little irregularities in the cob size, 4 = ears with fully filled grains, disease free with little insect damage and few irregularities in the cob size, 5 = ears with fully filled grains, mild disease and insect damage with few irregularities in the cob size, 6 = ears with smaller, non-uniform but fully filled cobs with severe disease and insect damage, 7 = ears with scanty grain filling, fewer ears, severe disease and insect damage and non-uniform cobs, 8 = very few ears with few scanty grain filling and severe insect and disease damage, and 9 = not more than one or no ears produced. After harvesting, the number of ears per plant (EPP) was calculated by dividing the total number of ears harvested in a plot by the total number of plants at harvest. Grain yield (Kg/ha) was computed from shelled grain weight per plot and adjusted to a 15% moisture content.

### 2.4. DNA Extraction and DArTseq Genotyping

The GBS protocol developed by Elshire et al. [25] was adopted for this study. Briefly, the genomic DNA of each of the 30 inbred lines was isolated from fresh leaves bulked from ten plants/inbred lines using the modified CTAB method of DArT (https://ordering.diversityarrry.com/files/DArTDNAisolation.pdf accessed on 20 June 2018). The DNA quality was determined through 0.8% gel electrophoresis, with the DNA concentration quantified by a Nano-drop spectrophotometer (Thermo Scientific, Wilmington, DC, USA). Subsequently, the genotyping was performed at BeCA-ILRI, Nairobi, Kenya using the Integrated Genomic Services and Support (IGSS) platform. Ninety-six samples were multiplexed per sequencing lane and the SNPs calling was performed with DArT’s proprietary software, DArTSoft as previously described [26]. Reads and tags from each sequencing result were anchored to the B73 reference genome [26]. In total, 47441 SNPs were generated from the DArTseq platform. Then, we applied the call rate of >80% and markers with more than 5% missing values were eliminated using the TASSEL software version 5.2.13 [27]. The minimum and maximum frequency were set at 0.05 and 0.95, respectively to obtain 7834 high-quality DArTseq SNP markers for the analysis.

### 2.5. Statistical Analysis

Each location by year combination was regarded as a test environment. Analysis of variance (ANOVA) was performed for each research condition (CHDS, HS, and TDS) to determine the effects of genotype (G), environment (E), and the interaction (GEI). Subsequently, a combined analysis of variance was conducted across the three test conditions. The ANOVA was performed using the general linear model (PROC GLM) of the Statistical Analysis System (SAS) via a random statement with a test option [28]. In the ANOVA for each as well as across the research conditions, replicates within environments and incomplete blocks within replicates × environments interactions were considered as random factors while the entries (hybrids) were regarded as a fixed factor. The entry means were adjusted for block effects, according to the lattice design [29] and means were separated using standard error of difference (SED). Subsequently, NCD II analyses were carried out on plot means for data from all the research conditions using PROC GLM in SAS and a random statement with a test option. The dissimilarity within the hybrid component was decomposed into different sets including male sets, female sets, and female × male interaction sets in the NCD II ANOVA. The mean squares of the respective interactions (that is the male set and female set and female × male interactions) with the environment were used to calculate the F-test for the male, female, and the female × male interaction whilst the mean squares attributable to the environment × female × male sets were tested using the pooled error mean squares. The general combining ability (GCA) was described as the main effect of the male sets and female set while the specific combining ability (SCA) was the female × male interaction effect [30]. The relative importance of GCA and SCA effects was determined by using the equation proposed by Baker [31] and modified by Hung and Holland [32] as:

$$\frac{2k_{GCA}^{2}}{\left(2k_{GCA}^{2}+2k_{SCA}^{2}\right)}$$

where $2k_{GCA}^{2}$ is the quadratic form (analogous to a variance component but depicting a fixed effect) derived from the mean square of the GCA effect and $2k_{SCA}^{2}$ is the quadratic form of SCA effects since the total genetic variation among single-cross progeny is equal to twice the GCA component plus the SCA component. The closer this ratio is to unity, the greater the predictability of a specific hybrid’s performance based on GCA alone.

A base index (BI) that incorporated superior grain yield, ASI, PLASP, EPP, EASP, and LDC was used to select the best and worst-performing hybrids under each stress condition [33]. The traits included in the BI were first standardized using the standard deviation of 1 with a mean of zero to minimize the effect of the different scales. The BI was calculated as:

$$BI=\left[\left(2\times GYs\right)+EPP-ASI-PLASP-EASP-LDC\right]$$

where *GY_s_* is the grain yield under stress, EPP is ear per plant, ASI is anthesis silking interval, PLASP is plant aspect, and LDC is stay-green/leaf death characteristic. A positive BI value indicated tolerance to the applied stress while a negative BI value indicated susceptibility [33].

The heterotic grouping method based on the GCA of multiple traits (HGCAMT) postulated by Badu-Apraku et al. [21] was used to assign the inbred lines into HGs under each and across the research conditions. The grouping was attained by standardizing the GCA effects of all traits with significant mean squares under the respective research conditions, with emphasis on eight traits employed under HS and CHDS (GY, ASI, EPP, EASP, PLASP, LF, TB, and LDC) and six traits under TDS (GY, ASI, EPP, EASP, PLASP, and LDC). Under each research condition, standardized GCA values of selected traits were converted to Euclidean distance estimates using PROC DISTANCE in SAS version 9.4 [28] and subjected to Ward’s minimum variance cluster analysis to generate a dendrogram, which assigned inbreds to clusters at 40% level of dissimilarity (R^2^ = 40%). The statistical model adopted for the HGCAMT method is given below:

$$\gamma =\overset{n}{\underset{i=1}{\sum }}\left[\frac{\left(Gi-gi\right)}{s}\right]+\varepsilon ij$$

where *Y* is the genetic value depicting relatedness among inbred lines based on the GCA of multiple traits (HGCAMT), *i* to n; *G* is the individual GCA effects of inbred lines for trait i; *g* is the mean of GCA effects across inbred lines for trait *i*; s is the standard deviation of the GCA effects of trait *i*; *ξij* is the residual of the model associated with the combination of inbred i and trait j.

Similarly, the DArTseq SNP data was converted to the Euclidean distance matrix, from which a dendrogram was constructed to depict HGs at 40% dissimilarity.

For each research condition (CHDS, HS, and TDS) the DArTseq based GD and the HGCAMT methods of heterotic grouping were evaluated to determine the most effective method. This was achieved by assembling all the 150 hybrids from the highest to the lowest based on GY under each and across research conditions and dividing the total number of hybrids for each method into two major groups, that is the inter-group and intra-group crosses. Then, the hybrids were ranked based on GY and divided into three groups. Group one (high-yielding hybrids) comprised the top 50 hybrids, group two (intermediate hybrids) consisted of hybrids ranked between 51 and 100, and group three (low-yielding hybrids) was composed of hybrids ranked between 101 and 150. The better classification method was chosen using the method of Fan et al. [34]. Additionally, breeding efficiency was calculated by using the average of the proportion of total inter-heterotic group hybrids from the high yielding inter-heterotic group hybrid plus the proportion of total low yielding intra-heterotic group hybrids as proposed by Badu-Apraku et al. [35]. Thus, breeding efficiency was estimated using the equation below:

$$BE=\frac{\left[\frac{\mathrm{NHYINTERGH}}{\mathrm{TNINTERGH}}\times 100+\frac{\mathrm{NLYINTRAGH}}{\mathrm{TNINTRAGH}}\times 100\right]}{2}$$

where BE is breeding efficiency, NHYINTERGH = number of high yielding inter-heterotic group hybrids, TNINTERGH is the total number of inter-heterotic group hybrids, NLYINTRAGH = number of low yielding intra-heterotic group hybrids, and TNINTRAGH = total number of intra-heterotic group hybrids.

Inbred and single cross testers were identified by using the criteria of Pswarayi and Vivek [36].

The least significant (LS) means for GY obtained from data combined across the research conditions was subjected to genotype main effects plus genotype by environment interaction effects (GGE) biplot analysis and the result was displayed as graphs showing (1) a “which-won-where” pattern to identify mega-environments, and (2) ranking of hybrids based on yield and stability [37]. The GGE-biplot was constituted using the first two principal components (PC1 and PC2) based on the model equation below:

Ŷij−Yj=λ1ξi1ηj1+λ2ξi2ηj2+εij

where *Ŷ_ij_* is the average yield of genotype *i* in environment *j*; *Y_j_* is the average yield-across genotypes in environment *j*; *λ_1_* and *λ_2_* are the singular values for PC1 and PC2 respectively; *ξ_i1_* and *ξ_i2_* are the PC1 and PC2 scores, for genotype-i; *η_j1_* and *η_j2_* are the PC1-and -PC2 scores, for environment *j*; *ε_ij_* is the error associated with the genotype-*i* in the environment.

## 3. Results

### 3.1. Genotypic Variation and General and Specific Combining Ability Analyses

Under each and across the research conditions, G, E, GEI, set (groups of five inbred lines with different genetic backgrounds, each serving as both a male in one set and as a female parent in another set), and set by E interactions were highly significant (*p ≤ 0.001*) for GY and other traits (Table 1 and Table 2). Partitioning the variation into GCA and SCA effects showed that both were significant for GY and other traits under the research conditions. Similarly, mean squares (MS) for these same sources of variation were highly significant across research conditions for GY and the majority of the traits (Table 1 and Table 2). Further decomposition of the hybrid components into male (set) (GCA-m), female (set) (GCA-f), and the female × male interaction (set) (SCA) MS revealed that GCA-male, GCA-female, and their interactions with the environment were significant for GY and other measured traits under each and across the research conditions (Table 1 and Table 2). The sets (GCA-m, GCA-f, and SCA) by environment interaction MS were also significant for GY and most other traits under all research conditions (Table 1 and Table 2).

### 3.2. Effects of General Combining Ability for Agronomic Traits

The inbred lines tested in this study displayed significant (*p* < 0.01 and 0.05) negative and positive GCA effects for GY and key secondary traits under the applied stresses (Supplementary Table S3a–d). Under CHDS, TZEI 135, TZEI 182, TZEI 17, and TZEI, 1496 displayed significant and positive GCA effects as males and females for GY while TZEI 417 had negative and significant GCA effects for GY when used as either a male or female parent (Supplementary Table S3a). Among these inbred lines, TZEI 135, TZEI 182, and TZEI 1496, as well as TZEI 528, TZEI 188, and TZEI 752, recorded significant and positive GCA effects for PLTHT as males and females whereas, TZEI 422, TZEI 242, TZEI 240, and TZEI 268 had significant and negative GCA effects as males and females for the same trait. For ASI, TZEI 272 recorded significant and negative GCA effects when used as a female but positive GCA effects when used as a male. The majority of the lines recorded negative female and male GCA effects for PLASP and EASP. Under HS, TZEI 135 and TZEI 10 had significant and positive GCA-female and GCA-male effects for GY while TZEI 417 displayed significant and negative GCA effects for GY as male and female (Supplementary Table S3b). TZEI 1013 and TZEI 7 had positive and significant GCA-female effects for GY while TZEI 17, TZEI 18, and TZEI 240 had positive and significant GCA-male effects for GY. TZEI 763 and TZEI 31 had negative GCA effects when used as males. Similarly, TZEI 135 displayed significant and negative male and female GCA effects for EASP while TZEI 417 had significant and positive GCA male and female effects for EASP. TZEI 135 and TZEI 182 showed significant and negative GCA effects for PLASP as females whereas TZEI 502 and TZEI 242 exhibited positive and significant GCA effects for the same trait when used as females. For LDC under HS, TZEI 1517 displayed significant negative GCA effects as a male and female (Supplementary Table S3b). Under TDS, TZEI 135 had significant and positive GCA-female and male effects for GY (Supplementary Table S3c) while TZEI 18 and TZEI 417 exhibited significant and negative GCA effects for GY as females. Significant negative GCA effects were observed for ASI when TZEI 17, TZEI 240, and TZEI 1517 were used as females and TZEI 135 as a male. For PLTHT, 20% of the lines displayed significant and positive GCA male and female effects with TZEI 188 and TZEI 135 recording the largest GCA male and female effects, respectively (Supplementary Table S3c). About 23% of the inbred lines had significant and negative GCA-female and male effects for PLTHT. GCA female and male effects were both negative and significant for PASP and EASP for TZEI 135. Across test conditions, TZEI 135 showed significant positive and negative GCA effects for GY and PLHT, and EASP and PASP, respectively as male and female (Supplementary Table S3d).

**Figure 1:**
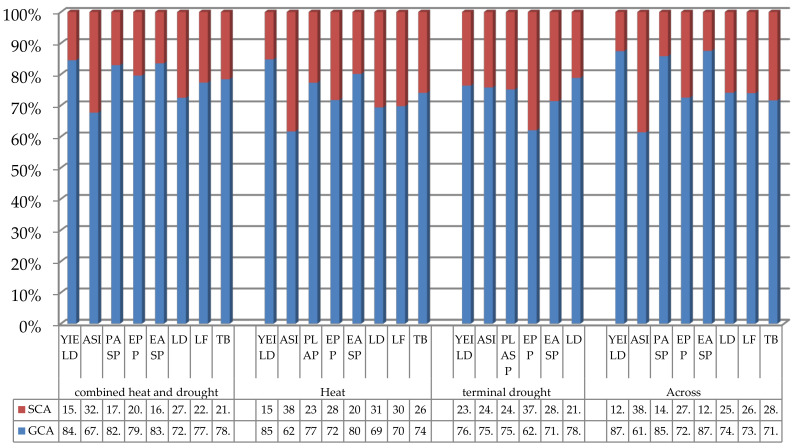
Proportion of GCA (lower bar) and SCA (upper bar) genetic variances for grain yield and other agronomic traits of tropical maize inbred lines under combined heat and drought, heat stress, terminal drought and across test environments. YIELD = Grain yield; SD= days to 50% silking; AD = days to 50% anthesis; ASI = anthesis-silking interval; PLHT = plant height; EHT = ear height; PASP = plant aspect; EPP = ears per plant; EASP = ear aspect; LD = leaf death; LF = leaf firing.

The proportion of MS of GCA and SCA relative to the total genetic variation among hybrids revealed that the contribution of GCA variances was larger than SCA variances for the measured traits (Figure 1). For instance, the proportion of GCA variances as compared to the total genetic variances among hybrids tested under CHDS ranged from 67.7% for ASI to 93.7% for PLHT. The SCA variance component varied from 6.4% (PLHT) to 32.3% (ASI) (Figure 1). A similar trend was observed under HS and TDS where the contribution of GCA variances to the total variation in traits ranged from 61.7–84.7% and 76.4–92% as compared to SCA variances that varied from 15.2–39.7% and 23.65 to 62.03%, respectively. This trend did not differ when data from all research conditions were combined. The importance of GCA and SCA variances was further tested using the equation proposed by Baker [31]. We observed very high (>0.60) ratios for all measured traits under each and across the conditions (Table 3), which confirmed the preponderance of GCA variances over the SCA.

### 3.3. Heterotic Grouping and Relationship among Inbred Lines

#### 3.3.1. Grouping Based on GCA of Multiple Traits

**Figure 2:**
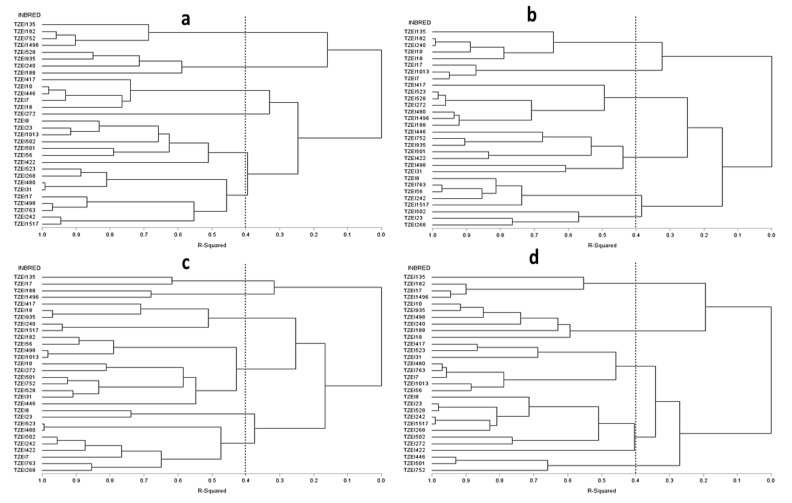
Dendrogram of 30 tropical maize inbred lines generated from Euclidean genetic distance-based GCA effect of grain yield and other traits (HGCAMT) using Ward’s minimum variance. (**a**) Under combined heat and drought environment (**b**) Under heat stress (**c**) Under terminal drought stress, and (**d**) Across environments. The imagery line indicate cut off point at 40% level of dissimilarity (R^2^ = 40%).

The dendrogram constructed using the HGCAMT method classified the 30 inbred lines into six HGs under CHDS, HS, and terminal DS conditions (Figure 2) at a 40% coefficient of determination. Based on BI values, the 22 inbred lines identified to be tolerant to CHDS were distributed across the six HGs. HG VI contained the highest number of inbreds (TZEI 268, TZEI 480, TZEI 31, TZEI 17, TZEI 498, TZEI 498, TZEI 768, TZEI 248, and TZEI 1517).

Similarly, all the 28 inbred lines identified with varying levels of tolerance to HS were distributed among the six HGs. A similar trend was observed for the 27 inbred lines identified to be tolerant to DS. Across test environments, the dendrogram generated using the HGCAMT method identified 5 HGs of inbred lines (Figure 2). The HGCAMT clustering method consistently placed TZEI 135 and TZEI 182 under HG I under CHDS, HS, DS, and across the three stresses.

#### 3.3.2. Grouping Based on DArTseq Marker Genetic Distances

**Figure 3:**
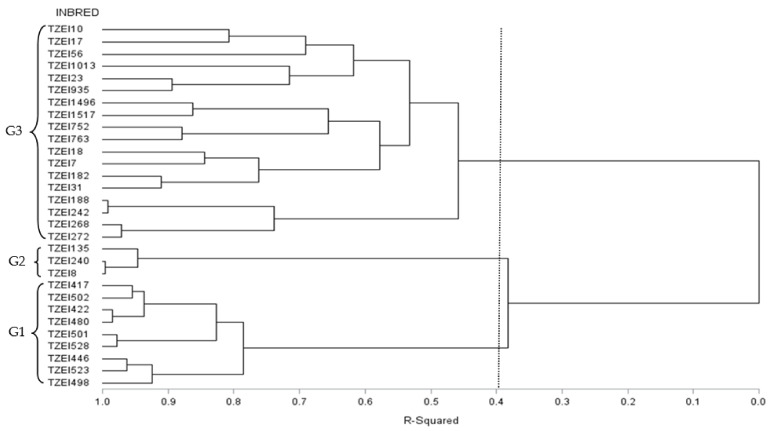
Dendrogram of 30 early-maturing maize inbred lines generated using cluster analysis of Euclidean genetic distance matrix obtained from Diversity Array Technology sequencing (DArTseq) markers. The imagery line indicates cut off point at 40% level of dissimilarity (R^2^ = 40%).

The dendrogram generated from the DArTseq genetic distance of the 30 inbred lines classified them into three major groups (G-1, G-2, and G-3) based on the pedigree, endosperm color, and reaction to the stresses (Figure 3). The first group consisted of nine inbred lines (TZEI 417, TZEI 502, TZEI 422, TZEI 480, TZEI 501, TZEI 528, TZEI 446, TZEI 523, and TZEI 498) with common ancestor/pedigree and endosperm colour. The second group contained three inbred lines, all tolerant to DS and CHDS. TZEI 240 and TZEI 18 had white endosperm color while TZEI 135 was yellow. Group 3 comprised four yellow and fourteen white endosperm inbred lines. The inbred lines TZEI 10, TZEI 182, TZEI 17, TZEI 23, TZEI 18, TZEI 188, TZEI 56, TZEI 7, TZEI 31, TZEI 242, TZEI 268, and TZEI 272 of group 3 recorded positive BI values and thus, were tolerant to CHDS and DS (Supplementary Table S1). The inbred lines TZEI 135 in group 2 as well as TZEI 10 and TZEI 17 in group 3 recorded significant and positive GCA-female and GCA-male effects for GY across environments (Supplementary Table S3d). Additionally, two inbred lines, TZEI 135 and TZEI 17 (with positive BI values), were highly tolerant to terminal DS.

### 3.4. Efficiency of Grouping Methods and Identification of Testers

Breeding efficiency based on HGCAMT and DArTseq GD methods ranged from 32% to 41.6% and 32% to 43.2%, respectively (Table 4). The DArTseq SNP-based GD method had the highest breeding efficiency of 43.2% under HS, 38.14% under TDS, and 39.2% across research conditions. Under CHDS, breeding efficiency computed from the HGCAMT method was 3.6% higher than that obtained from the DArTseq GD method. For both grouping methods (HGCAMT and DArTseq based genetic distance), the number of high-yielding inter-group hybrids was consistently higher for crosses obtained from parents of different HGs compared to the number of low-yielding intra-group hybrids (crosses within the same HG) under the test conditions (Table 4).

Pswarayi and Vivek, [36] demonstrated that the choice of lines as potential testers for classifying other lines into heterotic groups should be based on significant and positive GCA effects of grain yield, classification into heterotic groups, and high grain yield of inbred per se. Based on these criteria, TZEI 135 was identified as the best female and male inbred tester under HS, CDHS, TD, and across the contrasting environments. Similarly, TZEI 17 was identified as the best male and female tester under CDHS and across environments but only as a male tester under HS while TZEI 10 was identified as the best male and female tester under HS and across environments. Four single cross hybrids (TZEI 17 × TZEI 135, TZEI 135 × TZEI 182, TZEI 17 × TZEI 8 and TZEI 10 × TZEI 17), one from each test condition had their parental lines placed in similar HGs using the DArTseq based marker method. Moreover, the parental lines of these hybrids recorded significant and positive GCA-female and male effects for GY under all test environments. Amongst the five selected single cross hybrids, TZEI 10 × TZEI 17 recorded a relatively higher GY under HS and was identified as a potential early maturing yellow single cross tester.

### 3.5. Performance of Hybrids in Test Environments

Results of the top 20 and worse 10 hybrids based on index selection under the research conditions are presented in Supplementary Table S4. Under CHDS, GY varied from 405 to 1274 kg/ha with a mean of 838.84 kg/ha. The top 15 hybrids significantly out-yielded the best check by 5–17% (Supplementary Table S4a). In contrast, GY under HS varied from 650 to 8239 kg/ha with an average of 3715 kg/ha. The best hybrid (TZEI 1013 × TZEI 240) produced 46% more GY than the best commercial hybrid (TZEI 86 × TZEI 60) (Supplementary Table S4b). Under TDS, GY averaged 1286 kg/ha from a variance of 214 to 4580 kg/ha. TZEI 17 × TZEI 8 was identified as the best hybrid with a yield advantage of about 70% over the best commercial check (Supplementary Table S4c). Across research conditions, GY averaged 1383 kg/ha. The GY of the top 3 hybrids significantly exceeded the grain yield of the best commercial check (TZEI 86 × TZEI 60) by between 5 and 21% (Supplementary Table S4d). Generally, the best hybrids under each and across the test environments were characterized by increased EPP, desirable PLASP, EASP, and HC with good stay-green characteristics.

### 3.6. Stability of Hybrid Performance across Research Conditions

**Figure 4:**
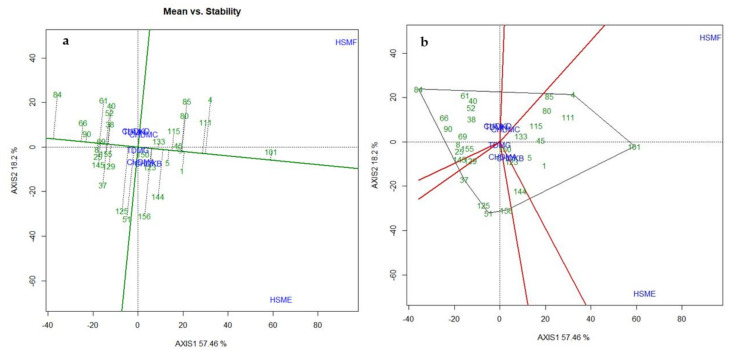
The “mean vs. stability” (**a**) and the“which-won-where” (**b**) hybrid plus hybrid × environment interaction biplot constructed based on grain yield of the top 20 and worst 10 hybrids including the checks under CHDS, HS, and terminal drought environments during the 2018 and 2019 dry seasons in Ghana and Nigeria.

From the GGE-biplot analysis, the first two axes (Axis 1 and Axis 2) together explained 75.66% of the variation in GY of the hybrids (Figure 4A). The polygon view (Figure 4B) of the GGE-biplot was used to show which hybrid performed best in which environments/locations. In the polygon view, the hybrid(s) that fell at the vertex of each sector of the biplot was the top-yielding hybrid(s) in that sector while those located closer to the origin of the biplot were considered to be more stable than the vertex hybrid. Hybrids located at the vertex of each sector were more responsive to their location as compared to those found closer to the origin of the biplot. Six single cross hybrids (entries 4, 101, 156, 51, 37, and 84) were found at the vertex of the polygon view (Figure 4B).

Two environments, (heat stress in Manga, 2018 and heat stress in Manga, 2019 (HSME and HSMF)) fell within the sector that had TZEI 135 × TZEI 501 and TZEI 240 × TZEI 1517 (Figure 4B; Supplementary Table S5) as the vertex hybrid, implying that these two early maturing hybrids were the best hybrids in terms of yield in these environments. However, the remaining environments were clustered close to the origin of the polygon suggesting that the environments were non-discriminating for grain yielding ability. Entries 156, 84, 37, and 51 were vertex hybrids but were not identified with any environments. Entry 150 was the least responsive hybrid to environmental variability.

The best hybrid should always demonstrate high mean yield performance as well as stability across test environments. In this study, the “mean performance vs. stability” GGE-biplot view was used to identify the highest yielding and most stable single cross hybrid across the test environments (Figure 4). The thick single-arrowed green line that passes through the biplot origin (intercept of the vertical and the horizontal broken axes) is known as the average-tester coordinate axis (ATC). Hybrids that yielded below the average yield (those to the negative side of the line) were separated from the hybrids above the average yield (those to the positive side of the line) by the thick vertical green line (ATC ordinate). Therefore, the grain yielding ability of a particular hybrid was measured by an imaginary projection from the position of the hybrid onto the ATC ordinate axis, while the stability was determined by the length of the projection onto the average tester coordinate axis. The farther away the hybrids were from the ATC ordinate (axis) in the right direction, the higher the GY. In contrast, the shorter the length of the projection of the hybrid to the ATC abscissa, the more stable was the hybrid. Based on these criteria, the entry/tester GGE biplot analysis identified TZEI 240 × TZEI 1517 (entry 101) as the highest yielding and most stable hybrid across the test environments (Figure 4).

## 4. Discussion

The combining ability of the 30 early maturing inbred lines was investigated in the present study under TDS, HS, and CHDS to obtain information on target agronomic traits for devising breeding strategies to improve the traits. An important requirement for the classification of germplasm into distinct HGs is the availability of adequate genetic variability. In the present study, the NCD II-derived single cross hybrids and their parental inbred lines displayed a wide range of variation for the traits measured under the test environments. In particular, the genotypic variations due to hybrids were highly significant for GY and some other traits, indicating the presence of adequate genetic differences among the parental lines and their derived hybrids. This implied that superior hybrids and promising lines could be identified for improvement through selection under the stresses. These results are consistent with the findings of previous studies which reported significant genetic variation among maize germplasm evaluated under similar stress conditions [7,8]. The effect of environment was also significant for GY and other measured traits indicating that each test environment was unique. Additionally, the observed significant GEI variances for the majority of the traits, indicated that the hybrids differed in their response to the varying environmental conditions tested in this study. The implication is that to ensure progress in varietal improvement, genotypes should be tested in multiple environments and different genotypes should be selected for specific growing environments. We found that under HS, CHDS, and across research conditions, variance due to genotypes and their interactions with environments were not significant for an important secondary trait such as ASI. This implied that in this set of hybrids, the inclusion of ASI in index selection for increased yield potential under HS, CHDS, and across test conditions was not essential. This is consistent with the results of Badu-Apraku and Fakorede [2] and Obeng-Bio [19], but contradicted the results of several authors who reported ASI as an important trait for indirect selection for increased GY under drought-stressed environments [8,19,33,38,39].

The two complementary methods used to assess the importance of GCA and SCA variances both showed that GCA variances were larger than the SCA variances for all measured traits (Figure 1, Table 3), indicating the predominance of additive gene action over the non-additive. The predominance of additive gene action over the non-additive gene action has been demonstrated in tropical maize [10,19,21,40,41,42]. The implication of this observation is that population improvement methods that capitalize on additive gene action such as the S_1_ family selection, full-sib family, and half-sib family methods should be adopted to accelerate progress in the development of superior hybrids for DS and HS prone environments. The inbred lines with significant and positive GCA effects for EPP and GY are expected to contribute favorable alleles to EPP and GY while those with significant and negative GCA effects would contribute favorable alleles for ASI, PLASP, and EASP. Four inbred lines (TZEI 182, TZEI 1496, TZEI 10, and TZEI 17) had positive GCA-female and GCA-male effects for GY under CHDS, HS, and across test environments making them suitable for use as either female or male parents in the development of hybrids tolerant to the stresses. Similarly, under HS, the significant and positive GCA-female effects recorded for GY in TZEI 1013 and TZEI 7 suggested that these inbred lines could be important donors of beneficial alleles for enhanced GY potential when used as female parents. The same explanation holds for inbred lines TZEI 17, TZEI 18, and TZEI 240, which showed significant and positive GCA-male effects for GY under HS. Interestingly, inbred line TZEI 135 consistently displayed significant and positive GCA effects for GY and EPP and negative and significant GCA effects for PLASP and EASP, as both male and females, making it the best combiner for the four important traits in the favorable direction (increased EPP and GY and desirable PLASP and EASP). It is striking to note that our study identified nine inbred lines as good combiners (lines with significant and negative male and female GCA effects) for plant height in environments where shorter hybrids were typically preferred to minimize lodging. The larger GCA-female effects over the GCA-male for traits such as LDC, LF, and TB under HS and LDC under DS revealed that maternal effects played a major role in the inheritance. Thus, to take advantage of possible cytoplasmic inheritance, the inbred lines with significant and negative GCA-female effects for these traits should be used as female parents in their respective crosses. Additionally, TZEI 17 and TZEI 1517 with significant and negative GCA-female and GCA-male effects for LDC under HS, and TZEI 242 showing negative GCA–female and male effects for TB and LF under CHDS when used as either female or male parents, could contribute favorable alleles for delayed senescence leading to increased photosynthesis and ultimately, higher GY.

The 30 inbred lines analyzed in this study were also classified into HGs using two different grouping methods, namely the HGCAMT method postulated by Badu-Apraku et al. [21] and the DArTseq marker genetic distance method. High BE was detected by both methods confirming that the inbred lines involved in the hybrid combinations were genetically diverse. However, a comparison of the two methods revealed that the BE of the latter was higher under HS, TDS, and across research environments but not under CHDS. Similar results were reported by Lanza et al. [43], Badu-Apraku et al. [44], and Obeng-Bio [19] who reported relatively higher BE values for the molecular marker genetic distance method than the HGCAMT method under multiple stress environments. On the contrary, and Badu-Apraku et al. [35] and Akinwale et al. [45] showed that the HGCAMT method was more efficient in grouping tropical maize inbreds into HGs than the SNP-based grouping method under low-N, DS, and *Striga* infested environments. The DArTseq marker-based HG method grouped the 30 inbreds into three groups, consistent with their pedigrees and endosperm colors. Interestingly, all inbred lines in HG II were tolerant to both DS and CHDS. Thus, these inbred lines could be crossed to others in the opposing HGs to generate high-yielding hybrids for DS and CHDS-prone environments [19,34,46,47,48]. The inbred lines assigned to the same HG could be recombined to form different base populations that could be improved through various recurrent selection methods.

Pswarayi and Vivek [36] recommended the following criteria for the identification of effective inbred testers (i) the best tester should display significant positive GCA effects, (ii) it should belong to a distinct HG, and (iii) it should have the highest per se GY in its group. Using these criteria, inbred TZEI 135 was identified as the best female and male inbred tester under each and across the environments when the inbreds were grouped using DArTseq markers. Thus, TZEI 135 could be used as a parent in the development of high-yielding hybrids and synthetics when crossed with other inbreds from different HGs. Similarly, TZEI 17 was identified as a tester under CHDS and across environments while TZEI 10 was identified as a tester under HS and across the environments. Badu-Apraku and Akinwale, [49] had earlier identified TZEI 17 as the best inbred parent under DS, and TZEI 10 as the best under low-N conditions. The inbred TZEI 135 which was identified as the best male and female tester in this study could be used to group a large number of newly developed early maturing inbreds in the IITA-MIP yet to be field-tested.

In the present study, the average GY recorded for NCD II generated hybrids under HS (3715 kg/ha) was higher than that reported by Nelimor et al. [8] when a set of the early maturing tropical maize accessions were evaluated under HS. Outstanding hybrids under each stress condition were identified using a base index (BI) that integrated increased GY under each stress PLASP, EPP, short ASI, and delayed leaf senescence [33]. The base index indicated the response of hybrids to HS, DS, and CHDS [8]. A positive base index value indicated tolerance to the stress while a negative BI indicated susceptibility to the stress. Using the base index values under each stress, TZEI 56 × TZEI 1517 and TZEI 18 × TZEI 752 were identified to be among the best hybrids with positive BI (tolerant hybrids) under CHDS, which significantly out-yielded the best commercial hybrid check (TZEI 86 × TZEI 60). Four hybrids (TZEI 1496 × TZEI 242, TZEI 480 × TZEI 135, TZEI 135 × TZEI 182, and TZEI 56 × TZEI 1517) were identified as tolerant to both HS and TDS. The hybrids identified as tolerant to each of the stresses combined high GY potential with desirable agronomic characteristics could be good candidates for commercialization in SSA after further testing. The hybrids TZEI 56 × TZEI 1517, TZEI 135 × TZEI 182, and TZEI 1496 × TZEI 242 were tolerant to CHDS, HS, and TDS. These top three hybrids had a GY advantage of 5–21% over the best commercial hybrid check across test environments. These hybrids could be used as bi-parental crosses for pedigree selection to extract superior inbred lines for the development of multiple stress-tolerant hybrids. Furthermore, our study identified TZEI 10 × TZEI 17 as a potential single cross hybrid tester that could be used for the development of early maturing three-way hybrids for DS and/or HS environments.

Genotypic adaptation and stability in grain yield are two important attributes constrained by the interaction between genotype and environment (GEI), which is a major factor responsible for the variation in the performance of genotypes in contrasting environments. Among the objectives of this study was to identify hybrids that combined good yield and stable performance across a range of production environments for further testing for possible release and commercialization in the sub-region. The presence of significant GEI effects for GY and the majority of the measured traits under each and across the test environments indicated that the hybrids differed in their GY performance in the contrasting test environments. Consequently, the “which-won-where” and the “mean performance vs. stability” GGE biplot view were used to identify hybrids with location-specific and broad adaptation, respectively for GY. The first and second axis (Axis 1 and Axis 2) from the “which-won-where” view of the GGE biplot explained 75.66% of the variation in GY suggesting that Axis 1 and Axis 2 adequately estimated the environment-centered data. Entries 101 (TZEI 135 × TZEI 501) and 4 (TZEI 240 × TZEI 1517) showed specific adaptation to environments HSME (heat stress in Manga, 2018) and HSMF (heat stress in Manga, 2019) for high GY. Based on the “mean performance vs. stability” GGE biplot analysis, hybrids 101 (TZEI 135 × TZEI 501) and 45 (TZEI 501 × TZEI 528) were identified as the most stable hybrids across the test environments with entry 101 (TZEI 135 × TZEI 501) as the best hybrid because of its high GY across the test environments. This hybrid should therefore be extensively tested in on-farm trials to confirm the consistency of the performance and stability for commercialization in SSA.

## 5. Conclusions

The results of the present study showed that additive gene action was predominant over the non-additive for GY and most measured traits, suggesting that the GCA component largely accounted for the differences among the inbred lines. The DArTseq genetic distance-based method was more efficient in grouping the inbred lines and was used to identify TZEI 135 as the best tester under each and across test environments. The single cross hybrid TZEI 10 × TZEI 17 was identified as the best tester under HS while TZEI 135 × TZEI 182 was the best across environments. The most stable and high yielding hybrid across environments was TZEI 135 × TZEI 501 and should be tested in on-farm trials to confirm its consistency in performance for possible commercialization in SSA.

## Figures and Tables

**Table 1:** Mean squares from combined analysis of variance of hybrid maize evaluated under combined heat and drought, and heat stress environments at Manga and Kadawa during the 2017/2018 dry season.

Source	^a^ DF	^b^ YIELD (kg/ha)	^d^ SD (Days)	^e^ ASI (Days)	^j^ PLASP (Scale:1–9)	^k^ EASP (Scale:1–9)	^n^ EPP	^p^ LF (%)	^q^ TB
Combined Heat and Drought Stress									
^1^ Env	3	12,331,663.3 ***	3859.85 ***	1535.63 ***	261.49 ***	336.88 ***	0.09 **	0.21 ***	0.41 ***
Set	5	3,434,567.8 ***	59.27 ***	11.34	2.75 ***	7.85 ***	0.15 ***	0.15 ***	0.39 ***
Env * set	15	297,357.4 ***	43.38 ***	12.72 *	3.29 ***	4.85 ***	0.17 ***	0.02 *	0.02
Genotype	155	465,564.8 ***	18.73 ***	7	1.03 ***	1.21 ***	0.07 ***	0.02 ***	0.04 ***
Male (set)	24	486,510.5 ***	28.05 ***	6.19	1.54 ***	1.16 ***	0.09 ***	0.03 ***	0.05 ***
Female (set)	24	729,472.5 ***	24.23 ***	7.92	1.71 ***	2.11 ***	0.09 ***	0.02 **	0.04 **
Female * male (set)	96	222,641.04 ***	11.51	6.74	0.67 ***	0.64 ***	0.04 ***	0.01 *	0.02
Genotype * env	465	189,430.7 ***	13.86 ***	7.11	0.72 ***	1.04 ***	0.06 ***	0.01	0.02
Env * male (set)	72	167,949.31 ***	14.15 *	6.71	0.7 ***	0.71 ***	0.05 ***	0.01	0.02
Env * female (set)	72	247,017.7 ***	18.09 ***	6.39	0.89 ***	1.21 ***	0.07 ***	0.01 ***	0.03 *
Env * female * male (set)	288	162,849.2 ***	10.5	7.03	0.54 **	0.65 ***	0.05 ***	0.01	0.02
Error	522	7707.2	10.43	6.76	0.41	0.42	0.02	0.01	0.02
Heat Stress									
Env	1	872,905,949.1 ***	11574.18 ***	1196.44	22.19 ***	61.97 ***	0.76 ***	0.05	0.38
Set	5	19,992,488.1 ***	55.95 ***	423.37	2.94 ***	4.21 ***	0.16 ***	0.03 *	0.09
Env * set	5	3,670,697.2 ***	6.17	424.03	1.98 ***	2.11 **	0.58 ***	0.02	0.21
Genotype	155	4,807,572.3 ***	15.94 ***	439.79	0.91 ***	1.39 ***	0.07 ***	0.18 ***	0.25 ***
Male (set)	24	7,941,325.8 ***	23.48 ***	391.07	0.83 ***	2.49 ***	0.06 **	0.01	0.15
Female (set)	24	7,003,187.1 ***	27.12 ***	387.34	1.49 ***	1.49 ***	0.07 ***	0.03 **	0.17
Female * male (set)	96	2,683,972.1 ***	10.07	484.01	0.68 ***	0.99 ***	0.05 ***	0.02	0.11
Genotype * env	155	2,878,702.1 ***	12.18 ***	436.93	0.700 ***	0.83 **	0.09 ***	0.19 ***	0.28 ***
Env * male (set)	24	2,070,038.2 ***	16.69 **	366.51	0.61 *	0.86	0.06 **	0.02	0.17 *
Env * female (set)	24	3,425,366.8 ***	8.49	371.51	0.69 **	0.94 *	0.07 ***	0.01	0.11
Env * female * male (set)	96	2,657,296.4 ***	11.03 *	488.09	0.64 ***	0.81 *	0.06 ***	0.02	0.12
Error	262	191,640	7.68166	437.585	0.38	0.59	0.03	0.05	0.13

*, **, *** = Significant at 0.05, 0.01, and 0.001 probability levels, respectively; ^1^ environment, ^2^ replication; ^a^ degrees of freedom, ^b^ grain yield, ^d^ days to 50% silking; ^e^ anthesis-silking interval, lodging; plant aspect; ^k^ ear aspect; ^n^ ears per plant, ^n^ ears per plan, ^p^ leaf firing, ^q^ tassel blasting, Set (groups of five inbred lines with different genetic backgrounds), Male (set) refers to groups of five inbred lines with different genetic backgrounds serving as male parents in one set; Female (set) is groups of five inbred lines with different genetic backgrounds that served as female parents in another set.

**Table 2:** Mean squares from combined analysis of variance of hybrid maize evaluated under terminal drought and across environments in Manga and Kadawa during the 2017/2018 dry season.

Source	^a^ DF	^b^ YIELD (kg/ha)	^d^ SD (Days)	^e^ ASI (Days)	^j^ PLASP (Scale:1–9)	^k^ EASP (Scale:1–9)	^n^ EPP	^p^ LF (%)	^q^ TB (%)
Terminal Drought Stress									
^1^ Env	1	8,547,258.20 ***	141.27 ***	0.0005	1552.78 ***	10.32 ***	1.24 **	788.57 ***	
Set	5	2,768,389.49 ***	24.87 ***	9.06 ***	3.97 ***	1.54 ***	0.27	0.88 **	
Env * set	5	546,048.98 *	1.18	2.05 *	0.41	0.14	0.14	0.78 **	-
Genotype	155	855,779.0 ***	5.58 ***	1.61 ***	0.63 ***	0.42 ***	0.21 *	0.42 ***	-
Male (set)	24	702,898.04 ***	10.13 ***	1.86 **	0.63 ***	0.36 **	0.15	0.51 **	-
Female (set)	24	1,442,564.23 ***	7.47 ***	1.69 **	0.71 ***	0.35 **	0.21	0.62 ***	-
Female * male (set)	96	663,950.50 ***	2.75 **	1.13	0.44 ***	0.29 **	0.22 *	0.31	-
Genotype * env	155	418,299.9 ***	2.03	1.27 **	0.37 **	0.26 *	0.17	0.37 **	-
Env * male (set)	24	348,748.92 *	1.86	1.50 *	0.36	0.26	0.15	0.48 **	-
Env * female (set)	24	581,963.92 ***	1.81	1.33	0.41 *	0.33 *	0.13	0.59 ***	-
Env * female * male (set)	96	345,575.29 **	2.01	1.18	0.32	0.21	0.19	0.24	-
Error	262	209,950.4	2	0.95	0.26	0.21	0.16	0.25	-
Across Test Environments									
^1^ Env	7	459,751,108 ***	11343.41 ***	1333.61 ***	387.24 ***	483.81 ***	1.83 ***	0.26 ***	0.39 ***
Set	5	13,383,275 ***	12.22	176.86	6.21 ***	7.36 ***	0.15 *	0.17 ***	0.44 ***
Env *set	35	2,502,667 ***	36.99 ***	104.84	2.28 ***	3.32 ***	0.24 ***	0.02 *	0.06
^2^ Rep(env * set)	40	104,024	7.09	50.14	0.42	0.68 **	0.08 *	0.01	0.07
Block(env * rep)	194	116,728	17.87 ***	109.61	0.71 ***	0.82 ***	0.07	0.01 **	0.07 *
Genotype	155	378,998,432 ***	16.59 ***	111.53	1.33 ***	1.47 ***	0.11 ***	0.08 ***	0.12 ***
Male (set)	24	3,849,768 ***	34.35 ***	97.13	1.79 ***	2.11 ***	0.09 *	0.03 ***	0.07
Female (set)	24	4,043,843 ***	24.33 ***	95.69	2.56 ***	2.73 ***	0.16 ***	0.03 ***	0.08 *
Female * male (set)	96	1,135,578 ***	10.26 *	121.45	0.72 ***	0.68 ***	0.09 ***	0.02 ***	0.06
Genotype * env	1085	1,1805,42,807 ***	11.31 ***	113.59	0.64 ***	0.83 ***	0.11 ***	0.07 ***	0.11 ***
Env * male (set)	168	1,220,148 ***	12.44 ***	99.54	0.61 ***	0.76 ***	0.08 ***	0.01	0.07 **
Env * female (set)	168	1,442,533 ***	14.24 ***	100.63	0.72 ***	0.88 ***	0.09 ***	0.01 **	0.06
Env * female * male (set)	672	847,896 ***	8.36	125.31	0.52 **	0.61 ***	0.09 ***	0.01	0.05
Error	1046	104,436	7.63	113.21	0.36	0.41	0.06	0.02	0.06

*, **, *** = Significant at 0.05, 0.01, and 0.001 probability levels, respectively; ^1^ environment, ^2^ replication; ^a^ degree of freedom, ^b^ grain yield, ^d^ days to 50% silking; ^e^ anthesis-silking interval, ^j^ plant aspect; ^k^ ear aspect; ^n^ ears per plant, ^p^ leaf firing, ^q^ tassel blasting; Set (groups of five inbred lines with different genetic backgrounds), Male (set) refers to groups of five inbred lines with different genetic backgrounds serving as male parents in one set; Female (set) is groups of five inbred lines with different genetic backgrounds that served as female parents in another set.

**Table 3:** Prediction of hybrid performance based on parental GCA values as measured by the ratio of $2k_{GCA}^{2}$ to $2k_{GCA}^{2}+2k_{SCA}^{2}$.

	GY (kg/ha)	SD (Days)	ASI (Days)	PLASP (Scale:1–9)	EASP (Scale:1–9)	EPP	LF (%)	TB (%)
Combined Heat and Drought Stress								
GCA	1,215,983	52.28	14.11	3.25	3.27	0.18	0.05	0.09
SCA	222,641	11.51	6.74	0.67	0.64	0.04	0.01	0.02
$\frac{2k_{GCA}^{2}}{\left(2k_{GCA}^{2}+2k_{SCA}^{2}\right)}$	0.845	0.82	0.677	0.829	0.836	0.818	0.833	0.818
Heat Stress								
GCA	14,944,513	50.6	778.41	2.32	3.98	0.13	0.04	0.32
SCA	2,683,972	10.07	484.01	0.68	0.99	0.05	0.02	0.11
$\frac{2k_{GCA}^{2}}{\left(2k_{GCA}^{2}+2k_{SCA}^{2}\right)}$	0.848	0.834	0.617	0.773	0.801	0.722	0.667	0.744
Terminal Drought Stress								
GCA	2,145,462	17.6	3.55	1.34	0.71	0.36	1.13	-
SCA	663,950.5	2.75	1.13	0.44	0.29	0.22	0.31	-
$\frac{2k_{GCA}^{2}}{\left(2k_{GCA}^{2}+2k_{SCA}^{2}\right)}$	0.764	0.865	0.759	0.753	0.71	0.621	0.785	-
Across Environments								
GCA	7,893,611	58.68	192.82	4.35	4.84	0.25	0.06	0.15
SCA	1,135,578	10.26	121.45	0.72	0.68	0.09	0.02	0.06
$\frac{2k_{GCA}^{2}}{\left(2k_{GCA}^{2}+2k_{SCA}^{2}\right)}$	0.874	0.851	0.614	0.858	0.877	0.735	0.75	0.714

**Table 4:** Breeding efficiencies of the heterotic group’s general combining ability of multiple traits and the DArTseq marker methods under combined heat and drought, heat, terminal, and across drought environments.

Yield Group	Cross Type	HGCAMT	DArTseq Markers
1	Inter	45	30
1	Intra	5	20
2	Inter	39	30
2	Intra	11	20
3	Inter	38	26
3	Intra	12	24
**Breeding efficiency (%)**		**39.8**	**36.2**
1	Inter	43	32
1	Intra	7	18
2	Inter	46	34
2	Intra	6	16
3	Inter	39	18
3	Intra	11	32
**Breeding efficiency (%)**		**41.6**	**43.2**
1	Inter	37	31
1	Intra	13	19
2	Inter	41	29
2	Intra	9	21
3	Inter	40	24
3	Intra	10	26
**Breeding efficiency (%)**		**31.3**	**38.14**
1	Inter	41	30
1	Intra	9	20
2	Inter	38	33
2	Intra	12	17
3	Inter	41	22
3	Intra	9	28
**Breeding efficiency (%)**		**32**	**39.2**

## Data Availability

The datasets used in the present study are available at the IITA CKAN repository.

## Supplementary Materials

The following supporting information can be downloaded at: https://www.mdpi.com/article/10.3390/plants11101365/s1, Table S1: Parental inbred lines involved in NCD II crosses to generate 150 single cross hybrids (a) and a description of commercial checks included during field evaluation of hybrids (b); Table S2: Average temperature and rainfall readings at Kadawa and Manga during the 2018 and 2019 cropping seasons; Table S3: Effects of general combining ability for grain yield and other agronomic traits of 30 early maturing tropical maize inbred lines assessed under combined heat and drought-stressed environments at Manga and Kadawa, 2017–2018 (a), heat stress at Manga, 2017–2018 (b), terminal drought-stressed environments at Manga (d) and across test conditions (d); Table S4: Grain yield and other agronomic traits of the 30 maize hybrids check inclusive (best = -0 and worst = 10) evaluated under combined heat and drought environments (3a) heat stress (3b), terminal drought (3c), and across test conditions (3d). Table S5: Description codes of hybrids and environments used for the GGE biplot analysis. Figure S1: (a) Mean temperature (minimum and maximum) at Manga during the flowering and grain filling periods during (April) the 2018/2019 cropping season. (b) Mean temperature (minimum and maximum) at Kadawa during the flowering and grain filling periods during (April) the 2018/2019 cropping season.

## References

[1] Byerlee D., Edmeades G. (2021). Fifty Years of Maize Research in the CGIAR: Diversity, Change and Ultimate Success.

[2] Badu-Apraku B., Fakorede M.A.B. (2017). Improvement of early and extra-early maize for combined tolerance to drought and heat stress in sub-Saharan Africa. Advances in Genetic Enhancement of Early and Extra-Early Maize for Sub-Saharan Africa.

[3] Daryanto S., Wang L., Jacinthe P.A. (2016). Global synthesis of drought effects on maize and wheat production. PLoS ONE.

[4] NeSmith D.S., Ritchie J.T. (1992). Effects of soil water-deficits during tassel emergence on development and yield components of maize (Zea mays L.). Field Crop. Res..

[5] Fahad S., Hussain S., Saud S., Khan F., Hassan S., Nasim W., Huang J. (2016). Exogenously applied plant growth regulators affect heat-stressed rice pollens. J. Agron. Crop Sci..

[6] Cairns J.E., Crossa J., Zaidi P.H., Grudloyma P., Sanchez C., Araus J.L., Menkir A. (2013). Identification of drought, heat, and combined drought and heat tolerant donors in maize. Crop Sci..

[7] Nelimor C., Badu-Apraku B., Tetteh A.Y., N’guetta A.S.P. (2019). Assessment of Genetic Diversity for Drought, Heat and Combined Drought and Heat Stress Tolerance in Early Maturing Maize Landraces. Plants.

[8] Nelimor C., Badu-Apraku B., Tetteh A.Y., Garcia-Oliveira A.L., N’guetta A.S.P. (2020). Assessing the Potential of Extra-Early Maturing Landraces for Improving Tolerance to Drought, Heat, and Both Combined Stresses in Maize. Agronomy.

[9] Prasad P.V.V., Djanaguiraman M., Perumal R., Ciampitti I.A. (2020). Impact of high temperature stress on floret fertility and individual grain weight of grain sorghum: Sensitive stages and thresholds for temperature and duration. Front. Plant Sci..

[10] Zaidi P.H., Zaman-Allah M., Trachsel S., Seetharam K., Cairns J.E., Vinayan M.T. (2016). Phenotyping for Abiotic Stress Tolerance in Maize Heat Stress: A Field Manual.

[11] Begna T. (2020). Combining ability and heterosis in plant improvement. Open J. Plant Sci..

[12] Acquaah G. (2012). Principles of Plant Genetics and Breeding.

[13] Brieger F.G. (1950). The genetic basis of heterosis in maize. Genetics.

[14] Lee M. (1995). DNA markers and plant breeding programmes. Adv. Agron..

[15] Akinwale R.O. (2021). Heterosis and heterotic grouping among tropical maize germplasm. Cereal Grains.

[16] Badu-Apraku B., Akinwale R.O. (2019). Biplot analysis of line × tester data of maize (Zea mays L.) inbred lines under stress and nonstress environments. Cereal Res. Commun..

[17] Badu-Apraku B., Fakorede M.A.B., Talabi A.O., Oyekunle M., Akaogu I.C., Akinwale R.O., Annor B., Melaku G., Fasanmade Y., Aderounmu M. (2016). Gene action and heterotic groups of early white quality protein maize inbreds under multiple stress environments. Crop Sci..

[18] Annor B., Badu-Apraku B. (2016). Gene action controlling grain yield and other agronomic traits of extra-early quality protein maize under stress and non-stress conditions. Euphytica.

[19] Obeng-Bio E. (2018). Genetic Analysis of Grain Yield and Other Traits of Early Maturing Provitamin A-Quality Protein Maize Inbred Lines under Drought and Low Soil Nitrogen Conditions. Ph.D. Thesis.

[20] Badu-Apraku B., Oyekunle M., Fakorede M.A.B., Vroh I., Akinwale R.O., Aderounmu M. (2013). Combining ability, heterotic patterns and genetic diversity of extra-early yellow inbreds under contrasting environments. Euphytica.

[21] Badu-Apraku B., Oyekunle M., Akinwale R.O., Aderounmu. M. (2013). Combining ability and genetic diversity of extra-early white maize inbreds under stress and nonstress environments. Crop Sci..

[22] Annor B., Badu-Apraku B., Nyadanu D., Akromah R., Fakorede M.A.B. (2019). Testcross performance and combining ability of early maturing maize inbreds under multiple-stress environments. Sci. Rep..

[23] Nasser L.M., Badu-Apraku B., Gracen V.E., Mafouasson H.N.A. (2020). Combining ability of early-maturing yellow maize inbreds under combined drought and heat stress and well-watered environments. Agronomy.

[24] Osuman A.S., Badu-Apraku B., Ifie B.E., Tongoona P., Obeng-Bio E., Garcia-Oliveira A.L. (2020). Genetic diversity, population structure and inter-trait relationships of combined heat and drought tolerant early-maturing maize inbred lines from west and central Africa. Agronomy.

[25] Elshire R.J., Jeffrey C., Glaubitz Q.S., Jesse A., Poland K.K., Edward S.B., Sharon  E.M. (2011). A robust, simple genotyping-by-sequencing (GBS) approach for high diversity species. PloS one.

[26] Kilian A., Sanewski G., Ko L. (2016). The application of DArTseq technology to pineapple. Acta Hortic..

[27] Bradbury P.J., Zhang Z., Kroon D.E., Casstevens T.M., Ramdoss Y., Buckler E.S. (2007). TASSEL: Software for association mapping of complex traits in diverse samples. Bioinformatics.

[28] SAS Institute Inc. (2017). SAS User’s Guide: Statistics.

[29] Cochran W.G., Cox G.M. (1960). Experimental Designs.

[30] Hallauer A.R., Miranda J.B. (1988). Quantitative Genetics in Maize Breeding.

[31] Baker R.J. (1978). Issues in diallel analysis. Crop Sci..

[32] Hung H.Y., Holland J.B. (2012). Diallel analysis of resistance to fusarium ear rot and fumonisin contamination in maize. Crop Sci..

[33] Badu-Apraku B., Oyekunle M., Akinwale R.O., Lum A.F. (2011). Combining ability of early-maturing white maize inbreds under stress and non-stress environments. Agron. J..

[34] Fan X.M., Zhang Y.M., Yao W.H., Chen H.M., Tan J., Xu C.X., Han X.L., Luo L.M., Kang M.S. (2009). Classifying maize inbred lines into heterotic groups using a factorial mating design. Agron. J..

[35] Badu-Apraku B., Fakorede M.A.B., Gedil M., Annor B., Talabi A.O., Akaogu I.C., Fasanmade T.Y. (2016). Heterotic patterns of IITA and CIMMYT early-maturing yellow maize inbreds under contrasting environments. Agron. J..

[36] Pswarayi A., Vivek B.S. (2008). Combining ability amongst CIMMYT’s early maturing maize (Zea mays L.) germplasm under stress and non-stress conditions and identification of testers. Euphytica.

[37] Yan W. (2001). GGE biplot: A windows application for graphical analysis of multi-environment trial data and other types of two-way data. Agron. J..

[38] Edmeades G.O., Bolaños J., Chapman S.C., Lafiite H.R., Bänziger M. (1999). Selection improves drought tolerance in tropical maize populations. I. Gains in biomass, grain yield and harvest index. Crop Sci..

[39] Ziyomo C., Bernardo R. (2013). Drought tolerance in maize: Indirect selection through secondary traits versus genome wide selection. Crop Sci..

[40] Derera J., Tongoona P., Vivek B.S., Laing M.D. (2008). Gene action controlling grain yield and secondary traits in southern African maize hybrids under drought and non-drought environments. Euphytica.

[41] Oyekunle M., Badu-Apraku B. (2014). Genetic analysis of grain yield and other traits of early-maturing maize inbreds under drought and well-watered conditions. J. Agron. Crop Sci..

[42] Adebayo M.A., Menkir A., Blay E., Gracen V., Danquah E., Hearne S. (2014). Genetic analysis of drought tolerance in adapted× exotic crosses of maize inbred lines under managed stress conditions. Euphytica.

[43] Lanza L.L.B., de Souza C.L., Ottoboni L.M.M., Vieira M.L.C., de Souza A.P. (1997). Genetic distance of inbred lines and prediction of maize single-cross performance using RAPD markers. Theor. Appl. Genet..

[44] Badu-Apraku B., Fakorede M.A.B., Oyekunle M., Akinwale R.O. (2015). Genetic gains in grain yield under nitrogen stress following three decades of breeding for drought tolerance and Striga resistance in early maturing maize. J. Agric. Sci..

[45] Akinwale R.O., Badu-Apraku B., Fakorede M.A.B., Vroh-Bi I. (2014). Heterotic grouping of tropical early-maturing maize inbred lines based on combining ability in Striga-infested and Striga-free environments and the use of SSR markers for genotyping. Field Crops Res..

[46] Smith O.S., Smith J.S.C., Bowen S.L., Tenborg R.A., Wall S.J. (1990). Similarities among a group of elite maize inbreds as measured by pedigree, F 1 grain yield, grain yield, heterosis, and RFLPs. Theor. Appl. Genet..

[47] Senior M.L., Murphy J.P., Goodman M.M., Stuber C.W. (1998). Utility of SSRs for determining genetic similarities an relationships in maize using an agarose gel system. Crop Sci..

[48] Betran F.J., Beck D., Bänziger M., Edmeades G.O. (2003). Genetic analysis of inbred and hybrid grain yield under stress and non-stress environments in tropical maize. Crop Sci..

[49] Badu-Apraku B., Akinwale R. (2011). Identification of early-maturing maize inbred lines based on multiple traits under drought and low N environments for hybrid development and population improvement. Can. J. Plant Sci..

