# Supplementary material for: Combining Ability and Heterotic Patterns of Tropical Early-Maturing Maize Inbred Lines under Individual and Combined Heat and Drought Environments
Source: Plants (Basel). 2022 May 20;11(10):1365. doi: 10.3390/plants11101365 (PMC9146004; doi:10.3390/plants11101365)
Supplement: Supplementary file 1 [file plants-11-01365-s001.zip › suplementary Figure S1.pdf]

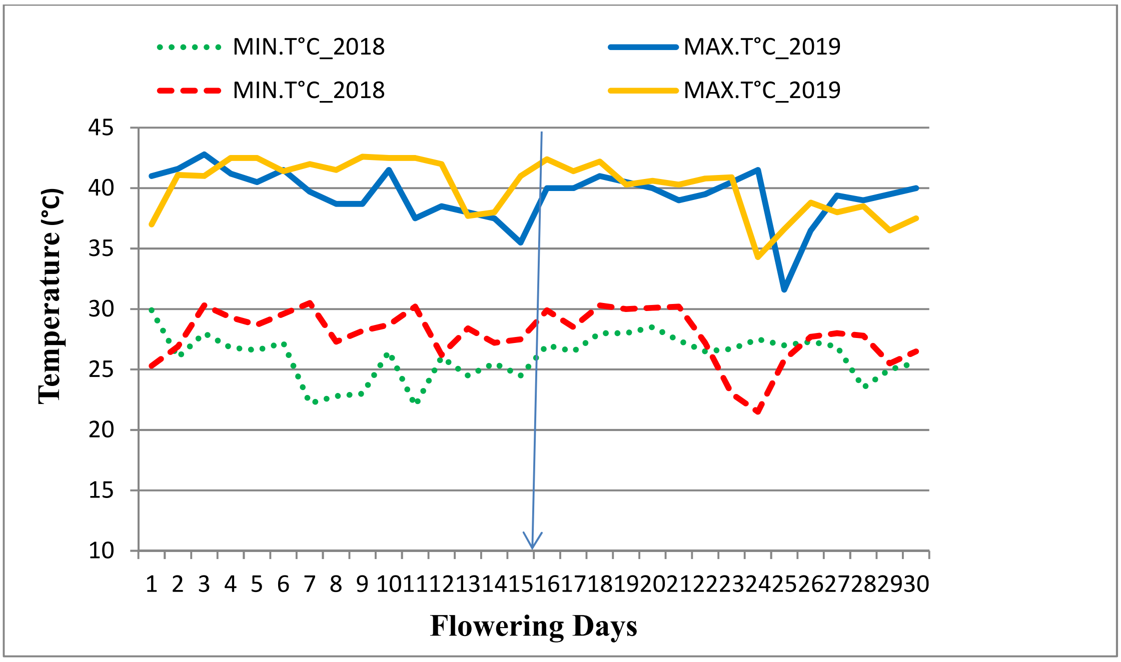

Supplementary figure S1a. Mean temperature (minimum and maximum) at Manga during the flowering and grain filling periods during (April) the 2018/2019 cropping season.

MIN= Minimum Temperature, MAX= Maximum Temperature, °C= Degrees Celsius

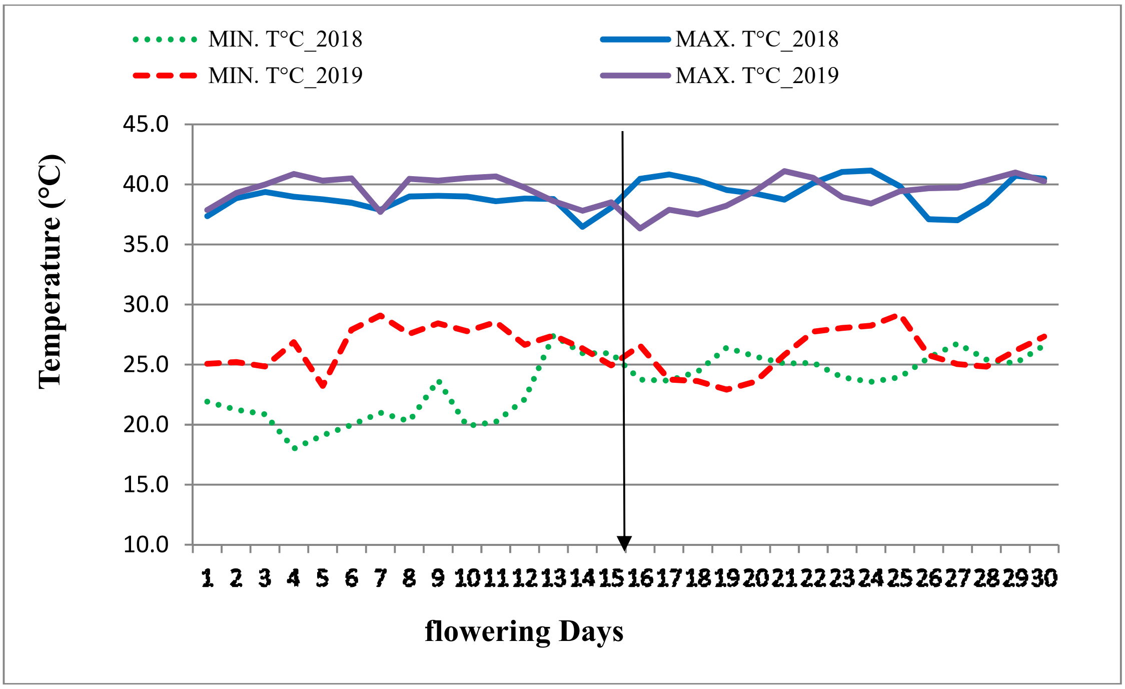

Supplementary figure S1b. Mean temperature (minimum and maximum) at Kadawa during the flowering and grain filling periods during (April) the 2018/2019 cropping season.

MIN= Minimum Temperature, MAX.= Maximum Temperature, ºC= Degrees Celsius
