# Supplementary material for: Combining Ability and Heterotic Patterns of Tropical Early-Maturing Maize Inbred Lines under Individual and Combined Heat and Drought Environments
Source: Plants (Basel). 2022 May 20;11(10):1365. doi: 10.3390/plants11101365 (PMC9146004; doi:10.3390/plants11101365)
Supplement: Supplementary file 1 [file plants-11-01365-s001.zip › Supplementary Table S5.pdf]

Supplementary Table S5. Description codes of hybrids and environments used for the GGE biplot analysis.

**Hybrid**	**code**
TZEI135xTZEI182	1
TZEI135xTZEI501	4
TZEI135xTZEI422	5
TZEI417xTZEI446	8
TZEI502xTZEI422	25
TZEI446xTZEI480	37
TZEI446xTZEI498	38
TZEI446xTZEI528	40
TZEI501xTZEI528	45
TZEI17xTZEI135	51
TZEI17xTZEI417	52
TZEI498xTZEI135	61
TZEI23xTZEI135	66
TZEI23xTZEI523	69
TZEI1013xTZEI272	80
TZEI18xTZEI268	84
TZEI18xTZEI272	85
TZEI763xTZEI272	90
TZEI240xTZEI1517	101
TZEI56xTZEI1517	111
TZEI56xTZEI935	115
TZEI272xTZEI31	123
TZEI272xTZEI935	125
TZEI1517xTZEI242	129
TZEI7xTZEI763	133
TZEI1496xTZEI242	144
TZEI1496xTZEI188	145
TZEI935xTZEI188	150
Check 5 - TZE-W Pop DT STR x TZEI 7	155
Check 6 - TZEI 86 x TZEI 60	156
**Environments**	**code**
Manga under Combined heat and drought stress, 2018	CHDMA
Kadawa under Combined heat and drought stress, 2018	CHDKB
Manga under Combined heat and drought stress, 2019	CHDMC
Kadawa under Combined heat and drought stress, 2019	CHDMD
Manga under HS, 2018	HSME
Manga under HS, 2019	HSMF
Manga under TDS, 2018	TDMG
Manga under TDS, 2019	TDMH
